# Scaling-up psychological interventions in resource-poor settings: training and supervising peer volunteers to deliver the ‘Thinking Healthy Programme’ for perinatal depression in rural Pakistan

**DOI:** 10.1017/gmh.2019.4

**Published:** 2019-04-26

**Authors:** N. Atif, A. Nisar, A. Bibi, S. Khan, S. Zulfiqar, I. Ahmad, S. Sikander, A. Rahman

**Affiliations:** 1 Human Development Research Foundation, Islamabad, Pakistan; 2 Health Services Academy, Islamabad, Pakistan; 3 Department of Psychological Sciences, University of Liverpool, Block B, Waterhouse Building, 1-5 Dover Street, Liverpool, Liverpool, UK

**Keywords:** Peer volunteers, perinatal depression, psychosocial intervention, task shifting, training and supervision

## Abstract

**Background:**

There is a scarcity of specialist trainers and supervisors for psychosocial interventions in low- and middle-income countries. A cascaded model of training and supervision was developed to sustain delivery of an evidence-based peer-delivered intervention for perinatal depression (the Thinking Healthy Programme) in rural Pakistan. The study aimed to evaluate the model.

**Methods:**

Mixed methods were employed as part of a randomised controlled trial of the intervention. Quantitative data consisted of the peers' competencies assessed during field training and over the implementation phase of the intervention, using a specially developed checklist. Qualitative data were collected from peers and their trainers through 11 focus groups during the second and third year of intervention rollout.

**Results:**

Following training, 43 peers out of 45 (95%) achieved at least a ‘satisfactory’ level of competency (scores of ⩾70% on the Quality and Competency Checklist). Of the cohort of 45 peers initially recruited 34 (75%) were retained over 3 years and showed sustained or improved competencies over time. Qualitatively, the key factors contributing to peers' competency were use of interactive training and supervision techniques, the trainer–peer relationship, and their cultural similarity. The partnership with community health workers and use of primary health care facilities for training and supervision gave credibility to the peers in the community.

**Conclusion:**

The study demonstrates that lay-workers such as peers can be trained and supervised to deliver a psychological intervention using a cascaded model, thus addressing the barrier of scarcity of specialist trainers and supervisors.

## Introduction

In recent decades, ‘task-shifting’ of psychosocial interventions, where non-specialists are trained to deliver such interventions under supervision of specialists, has emerged as a major strategy to meet the large treatment gap for common mental disorder in low- and middle-income countries (LMIC) (Eaton *et al*., 2011; Kakuma *et al*., 2011; Joshi *et al*., 2014). A number of randomised controlled trials (RCTs) from LMICs have established evidence for this approach (Rahman *et al*., 2013; Clarke *et al*., 2014; Fuhr *et al*., 2014). However, a major barrier to the scale-up of such interventions remains the lack of specialist health professionals to deliver training and supervision at scale. In most LMIC, primary care supervisors are often inundated with tasks perceived to be of higher priority such as nutritional advice, infection-control and immunisation, and do not have sufficient time or skills to provide such supervision (Jaskiewicz & Tulenko, 2012).

The Thinking Healthy Programme (THP) for perinatal depression (Rahman, 2007; Rahman *et al*., 2008) is an example of an evidence-based psychosocial intervention that can be delivered by non-specialists in community settings. To aid scale-up efforts through task shifting, we conducted a series of studies to evaluate the feasibility of peer-volunteers (local volunteer lay women who shared socio-demographic and life experiences with the target population) to work alongside community based lady health workers (LHWs) to deliver THP to their local communities (Singla *et al*., 2014). Following extensive formative work, THP was adapted and simplified for peer-delivery (Atif *et al*., 2017), and successfully piloted in rural Rawalpindi (Atif *et al*., 2016). An important component to ensure quality of intervention delivery, especially when deploying less skilled or unskilled workforce, is the adequacy of their training and supervision (Fulton *et al*., 2011). This paper describes the process of training and supervision of the peer volunteers in the Thinking Healthy Programme – Peer-delivered (THPP) using a cascaded model. Our aim was to use quantitative and qualitative methods to evaluate the training and supervision.

## Method

### Settings and participants

The study was embedded in a cluster RCT to evaluate the effectiveness of the THPP in rural Pakistan (Sikander *et al*., 2015). This two-armed cluster RCT was conducted in a sub-district of rural Rawalpindi with 11 union councils (the smallest rural administrative unit consisting of 10–15 villages of varying sizes), comparing THPP to Enhanced Usual Care in women experiencing perinatal depression. The current study evaluated the training and supervision process of the peer volunteers and was conducted in clusters where the intervention was implemented.

The economy of rural Rawalpindi is primarily agrarian-based, but about a third of people, mostly men, are engaged in non-farm jobs, such as working as unskilled or semi-skilled labourers, government employees or serving in the army. According to the Economic Survey of Pakistan (2013–2014), the female literacy rate is 34% as compared to the overall literacy rate of 57% in the general population. Each union council has a basic health unit (BHU), delivering Primary Health Care services to a population of about 25 000. It is staffed by a physician, a midwife, a vaccinator, 15–20 LHWs and their supervisor called Lady Health Supervisor. Each LHW is responsible for a community-cluster of approximately 1000 people or 150 homes, visiting five to seven homes daily.

We conducted extensive formative research to determine the type of peer volunteers that would be acceptable to the women and their families in rural settings of Pakistan (Singla *et al*., 2014). Key characteristics included being local, of child-bearing age, educated mothers with similar experiences to participants, good communication skills and good reputation in the community. They were identified with the help of LHWs who had intimate knowledge of the communities. LHWs, their supervisors and the THPP programme personnel interviewed potential peer volunteers. Forty-five peer volunteers were selected and offered training. The final selection was made following the successful completion of their classroom training.

Table 1 gives demographic characteristics of the peer volunteers. The majority were married, in their late 20s or early 30s, had at least 10 years of schooling and, on average, had two children.

**Table 1.** Characteristics of peer volunteers

Characteristics	Peer volunteers (*n* = 45)
Age (mean, s.d.)	30 (5.7)
18–25	7 (15.6%)
26–35	29 (64.4%)
36–45	9 (20%)
Years of education (mean, s.d.)	12 (2.1)
Primary	0(0%)
Middle	0(0%)
Secondary	22 (49%)
Intermediate	9 (20%)
Graduate	14 (31%)
Years of work experience (mean, s.d.)	0(0)
Married	33 (73.3%)
Single	10 (22.2%)
Divorced	2 (4.5%)
No of children (mean, s.d.)	2 (2.0)

### An overview of the cascade model of training and supervision

**Fig. 1.**
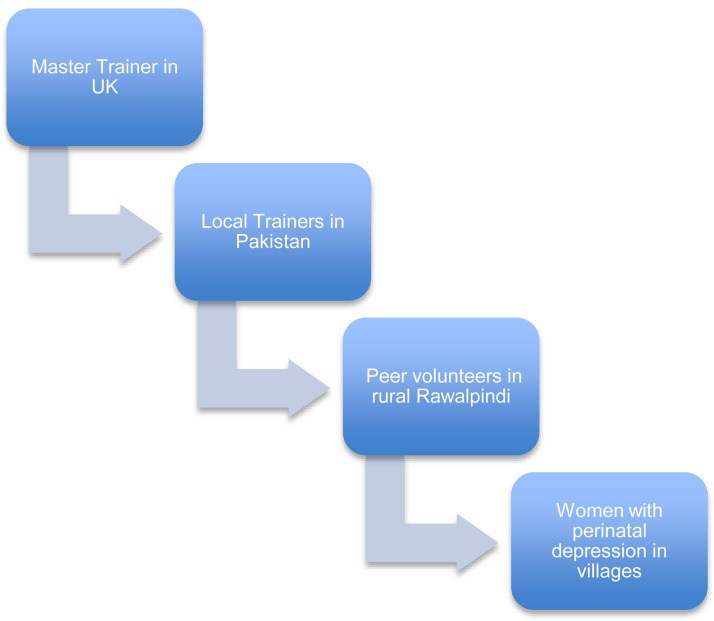
THPP cascade model of training and supervision.

A cascade model of training and supervision was employed for the peer-delivered programme. This model used a master trainer [mental health expert with cognitive behaviour therapy (CBT) training and in-depth understanding of the THP] based in the UK, to train and supervise the local THPP trainers (non-specialist university graduates in health or social sciences) based in a non-governmental organisation in Rawalpindi, Pakistan, who then trained and supervised the peer volunteers in their rural settings (Fig. 1). The trainings and supervisions were conducted at the BHUs, and facilitated by the Lady Health Supervisors.

#### Training of the local trainers

The master trainer, based in the UK, was trained in CBT, and had over 10 years clinical experience of working in mental health care. The local trainers consisted of five female university graduates with at least a bachelor's degree in health or social sciences and no prior mental health work-experience. The THPP training involved both classroom and field training and was cascaded down from the master trainer to local trainers to the peer volunteers (Fig. 1). Trainers received 20 h of classroom training by the master trainer in Pakistan, followed by 6 months of field training, which was supervised by the master trainer from distance. The classroom training focused on understanding of perinatal depression, use of counselling skills, CBT approach, key principles, contents and delivery mechanisms of THPP. In order to equip the trainers for their role, emphasis was laid on developing their training and supervisory skills along with assessing and addressing any potential risk. Training was delivered using power point presentations, group discussions, activities and role-plays. The field training, during which the trainees gained firsthand experience of delivering the intervention, followed the classroom training. During field-training each trainer delivered the intervention to at least two depressed mothers, located in the area contextually similar to study area, over a period of 6 months. During this period they received fortnightly supervisions by the master trainer.

#### Training of the peer volunteers

The training manual was developed with the aim of standardising the THPP training procedures. In total, five classroom trainings were conducted at five BHUs. The trainings conducted by the local trainers were spread over 5 days (approximately 30 h). The classroom training aimed to: (a) educate the peer volunteers on psychosocial factors impacting mother and child health during the perinatal period, (b) learn and practice basic counselling skills and (c) understand the intervention principles, contents and its delivery mechanisms. This mirrored the training they received from the master trainer. Different training methods were used, including lectures, discussions and activities, use of case scenarios, sharing personal experiences and role-plays. The role-plays focused on counselling skills, skills to engage mothers and their families during session delivery and dealing with challenging situations. The role-plays assessed peer volunteers' ability to deliver intervention. The intervention material consisting of the THPP reference manual and job-aids was given to the peer volunteers to assist them in the delivery of the intervention.

Following the classroom training, all peer volunteers deployed were assigned a mother (non-trial participants) to deliver the intervention for 3 months. This field training was aimed at providing the peer volunteers with the experience of practicing what they learned during the classroom training and to ensure their competencies for intervention-delivery before assigning them trial participants. The quality of the intervention and peer volunteers' competencies in delivering it were assessed through observing their individual sessions and group sessions and scoring them on the Quality and Competency Checklist developed for this purpose (see online Supporting Information file 1).

#### Supervision of the local trainers

The local trainers received their supervisions fortnightly from the master trainer. These supervisions were conducted via Skype and lasted between 60 and 90 min. They were mainly focused on challenges experienced by the trainers and peer volunteers, exploring problem-solving strategies and addressing any work-related stresses.

#### Supervision of the peer volunteers

Peer volunteers received both group and individual supervisions from the trainers. Five group supervisions were conducted each month at peer volunteers' respective village clusters. In order to standardise the supervision procedure, a supervision guide was developed outlining the key areas for supervision. These included: (1) exploring the peer volunteers' experience of delivering the sessions, (2) discussing challenges and how best to deal with them, (3) revising the content of the THPP and practicing through role-plays and (4) sharing the success stories and ensuring peer volunteers' motivation and wellbeing. Salient issues emerging from these supervision sessions were highlighted on the supervision forms by the trainers and discussed with the master trainer.

Individual supervision (called field supervision) involved the trainer accompanying the peer volunteer in the field to observe the delivery of a session. These sessions were aimed at: (a) assessing the peer's level of engagement with the mothers and their families, (b) assessing her competency of intervention delivery, (c) understanding fieldwork challenges and (d) providing performance feedback. The field supervision enhanced the credibility and reliability of peer volunteers' work and strengthened their links with the LHWs and the target communities.

### Evaluation of training and supervision

#### Study design

Mixed methods were employed to evaluate THPP training and supervision procedures. The qualitative study was embedded within the THPP trial phase and was aimed to understand the experiences of trainers and peer volunteers of delivering and receiving training and supervision. Interview guides were developed and pilot tested and data were collected through focus group discussions (FGDs). Focus groups were conducted by the four THPP trainers who were trained in qualitative methods. Each trainer conducted focus groups with a set of peers not trained or supervised by them. The focus group with the trainers was conducted by an independent researcher (IA), who was independent of the training and supervision processes. The peer volunteers and the local trainers’ focus groups were conducted at the BHU and research centre, respectively. Field notes were made during the focus group. Data were analysed using the framework analysis approach. All interviews were recorded, transcribed and familiarised, and themes were identified. The key themes derived were clustered under the categories of ‘facilitators’ and ‘barriers’ to the training and supervision, and a thematic framework was developed. Each theme and its sub-theme in the thematic framework were given an index number. The raw data were revisited and indexed. This was followed by charting, which involved summarisation of indexed sections from the raw data and placing them on thematic charts. All summaries included in the chart were referenced to allow an audit trail of the findings.

Quantitative data consisted of peer volunteers' competencies assessed during field training and at three further time points: time point 1 (6 months after training), time point 2 (12 months after training) and time point 3 (24 months after training).

#### Measurements

A Quality and Competency Checklist was specially developed to assess therapy quality and peer volunteers' competency to deliver the intervention on six areas with a number of items for each area. These areas included: (1) develops an empathetic relationship, (2) ensures family support and participation, (3) delivers the contents of the individual sessions, (4) uses effective problem-solving strategies, (5) conducts group session effectively and (6) deals with the challenging situations. The checklist was informed by ENhancing Assessment of Common Therapeutic factors (ENACT) an 18-items tool, used by the non-specialists for peer ratings of skills for delivering psychosocial interventions in low-resource settings (Kohrt *et al*., 2015). The evaluation was conducted by the trainers but to reduce bias, each trainer evaluated a set of peers not trained or supervised by them. The trainers observed the peer volunteers delivering a session and scored them on all items of the Quality and Competency Checklist. The items in each area were rated on a Likert scale (0–2) ranging from not demonstrated, partially demonstrated and demonstrated well, with an option of not applicable. Each area was separately scored and score was converted into percentage. A minimum score of 70% in each area indicated satisfactory competency. Peer volunteers scoring <60% were re-trained and those scoring <50% were not recruited. Peer volunteers who achieved overall satisfactory level of competency (70% or above on the total score for all five areas) were rated competent and assigned cases for intervention. Formal inter-rater reliability was not conducted but evaluators were trained by rating the same session concurrently and comparing their scores and discussing discrepancies with the master trainer.

## Results

### Retention and replacement of the peer volunteers

From the cohort of 45 peer volunteers initially recruited, 34 peer volunteers (75%) were retained over the course of the programme (April 2014–March 2017). In some cases a peer volunteer was replaced more than once over the trial phase, making a total of 21 replacements. The most common reason for the peer volunteers dropping out included changes in their personal circumstances (*n* = 9), scoring less than acceptable levels of competency (*n* = 6), moving out of the study area (*n* = 5) and not being acceptable to the target population (*n* = 1). The newly recruited replacement peer volunteers, following their classroom and field training, gained further work experience through shadowing another peer for at least 1 month. Following this, their competencies were assessed and upon achieving the satisfactory competency score (⩾70%), were assigned to the study.

### Peer volunteers' competencies to deliver THPP

The overall peer volunteers' competency scores were based on rating the peer volunteers' delivery of two individual sessions and one group session during their field training, using the Quality and Competency Checklist. Of 45 peer volunteers initially trained, 95% of the peer volunteers scored 70% or above. The peer volunteers' competencies were reevaluated following their training at three further time points: time point 1 (6 months after training), time point 2 (12 months after training) and time point 3 (24 months after training). Table 2 gives breakdown of peer volunteers' competencies, for individual competencies. Only the original cohort of 34 peer volunteers who were retained through the entire study is presented to demonstrate their progression over time. Based on the data obtained from this original cohort during these time points, the majority of the peer volunteers showed either sustained or improved level of competencies.

**Table 2.** Competency scores

Competency scores	Field training (*n* = 45)	Time point 1 (*n* = 34)	Time point 2 (*n* = 34)	Time point 3 (*n* = 34)
80% or above *Good level of competence*	24 (53.3%)	19 (56.0%)	28 (82.4%)	25 (73.5%)
70% to 79% *Satisfactory level of competence*	19 (42.3%)	12 (35.0%)	6 (17.6%)	9 (26.4%)
60% to 69% *Lacking in some areas of competence*	2 (4.4%)	3 (9%)	0	0
50% to 59% *Lacking in competence*	0	0	0	0
Less than 50% *Unacceptable level of competency*	0	0	0	0

### Attendance of group supervisions

In total, 125 group supervisions (25 in each study clusters) were conducted between November 2014 and March 2017. Each supervision session lasted on average 3.5 h. The overall attendance record in most centres was above 85% throughout 3 years, with some centres showing 100% attendance in year 3.

### Process evaluation of facilitators and barriers to training and supervision

In total, qualitative data were collected from peer volunteers and trainers through 11 focus groups. All peer volunteers and local trainers were included in the sample. Focus groups were conducted by the THPP trainers (AN, AB, SK and SZ). It was ensured that the trainers collected data from the group of peer volunteers, who were outside their cluster area and they were not involved in their training and/or supervision. The focus group, with the trainers, was conducted by the research fellow (IA), who was independent of the training and supervision processes for the delivery of the intervention. Initially five focus groups with peer volunteers (*n* = 41) during second year and later another five focus groups (*n* = 44) during third year and one focus group with the THPP trainers (*n* = 5) at the end of the trial. Each focus group lasted for up to an hour. All interviews were recorded and transcribed. Data collection and analysis was conducted simultaneously. Data were analysed using framework analysis by NA, AN, AB, SK, SZ and IA. In order to avoid biases during data analysis each transcript was analysed by two researchers, themes derived from the data were compared and in case of discrepancy field notes were referred and/or a third researcher was invited to code the data. The thematic framework indicating the key themes for the facilitators and barriers to training and supervision along with the quotes are presented in Table 3.

**Table 3.** Facilitators and barriers to the THPP training and supervision

Facilitators to the THPP training	
Ability to relate to the trainers	We never felt intimidated during the training. Whenever we asked any questions, whether relevant or not, trainers gave us answers very patiently and politely, which was encouraging for us. (Peer – FGD1) The problem-solving strategies the trainers suggested were appropriate to use within our context. They used to give examples, which helped me a lot in understanding how to address an issue. (Peer – FGD3)
Perceived usefulness of the training	Our training has made us real volunteers. It has given us confidence and motivation and has taught us how to engage with the mothers effectively. We learned so much from our training. (Peer – FGD1)
Training techniques	We have learnt a lot from our classroom training, the most through role-plays. They were really helpful. (Peer – FGD3) If a peer is still lagging in competency, we continue to train and assess her until desired competency is achieved. (Trainer – FGD11)
Linkage with the primary health system	Everyone (in the community) knows that LHWs are government employees and give useful information. When we tell them about our trainings at BHU and about our linkage with LHW, people show more respect to us, they know that we will also give them useful information. Without this linkage it would be difficult. (Peer volunteers – FGD7) BHU is an appropriate place (for trainings or supervisions) because it is located in the centre of our village and is acceptable for everyone. People value you more when they know you have received training from a BHU. (Peer – FGD4)
Increased psychosocial awareness and wellbeing	After THPP training, I started to observe myself (reflect). I changed my own unhealthy thoughts and behaviour. Now I have improved a lot in so many ways. (Peer – FGD5) That was the first time that I stepped out of my house on my own. Training helped me to gain confidence and to trust my abilities. (Peer – FGD5)
Barriers to the THPP training	
Lack of refresher trainings	Refresher trainings should be more often, so that we do not miss or forget anything related to our work. It should be conducted regularly after every 2–3 months. (Peer – FGD1)
Household commitments	I started feeling tense after mid-day. I wanted to leave, knowing that there is so much work to do at home. (Peer – FGD2)
Peer volunteers' no prior exposure to receiving trainings	I used to think that it would be a difficult work and I wouldn't be able to do it. I was very afraid before taking this job. (Peer – FGD4) Peers had their own reservations at the start of training. But with time they get used to it and were fine with us and with the training. (Trainer – FGD11)
Facilitators to the THPP supervision	
Experiential learning	When we attend our monthly supervision meetings, we discuss with each other success stories, challenges and issues about our work. It is very helpful and important because we get to learn from others' experiences. (Peer – FGD3)
Supervisor–supervisee relationship	Our supervisor's good communication skills helped us to overcome apprehensions of receiving supervision over Skype. She was open to suggestions and knew the intervention inside out. (Trainer – FGD11) If we experience any problem during field or any query, we ask during our group supervision meetings. They (trainers) always guide us in a very polite manner. (Peer – FGD9)
Practicing through role plays	Role-plays help us to practice. The feedback we receive from our supervisor and other peer volunteers reassured us that we are doing it right. (Peer – FGD9)
Improved confidence and self-esteem	I have always thought I am not good enough. During supervisions through sharing my experiences and receiving praise and encouraged, I started gaining confidence which is now spilling over to other parts of my life. (Peer – FGD8) I wasn't able to communicate with people easily, but now speaking in front of hundreds of people is not a problem for me. Who would have thought that one day I would become the lady counsellor for my area. (Peer – FGD4)
Barriers to the THPP supervision	
Challenges of using Skype	Most of the time Skype is good to communicate. However, sometimes the quality of the net is not good and we struggle to communicate, which is frustrating. Also while conducting supervisions via Skype we can miss on the non-verbal communication, which otherwise could have been useful. (Trainer – FGD11)
Lack of facilities at the BHU	Another barrier for group supervisions is lack of facilities at the BHU, sometimes there is no spare room, no rest-room or electricity available. At some centres, we are mostly conducting our supervision outside under a tree or in an open ground. (Trainer – FGD11)
Competing interests/responsibilities	Sometimes we feel that group supervisions are rushed because peer volunteers have to leave early because of their household commitments or its Friday or the BHU staff is waiting for us to leave to shut the centre. It also gets difficult during the harvesting season, as that is the time when peer volunteers are pre-occupied with cutting crops making it hard for us to engage them during supervision. (Trainer – FGD11)

## Discussion

The paper describes a cascade model of training and supervising peer volunteers for scaling-up of the THP in rural Pakistan. The key findings were that majority of the peer volunteers, upon receiving training from the non-specialist trainers, attained satisfactory level of competency to deliver the intervention, and showed improvement throughout the study period. The local non-specialist trainers felt equipped to take on their dual role, as trainers and supervisors, following training by the master trainer.

The cascade model used a tier of non-mental health specialist trainers between the mental health expert and local peer volunteers for cascading training and supervision. The model was successful as demonstrated by the finding that peer volunteers competencies increased over time with experiential learning and supervision. This is in line with evidence from other LMICs indicating cascaded training and supervision can be successfully implemented for delivering mental health care using lay workers (Murray *et al*., 2011; Gureje *et al*., 2015; Shields-Zeeman *et al*., 2017). The model used in the study, deployed the local trainers as both trainers and supervisors. The local trainers’ dual role had the advantages that they formed relationship with the peer volunteers during their training and supervise them with an in-depth understanding of the intervention. This also helped in overcoming some of the challenges presented by Murray's model such as supervisors' attrition, lack of understanding of the intervention and linguistic compatibility with the trainers.

There were a number of other facilitating factors that made the model feasible. The peer volunteers perceived the non-specialist local trainers to be accessible and approachable and they were able to discuss any work-related issues during supervisions. Using the primary care facility for the training and supervision, facilitated by the local LHWs, enhanced their credibility in the community, which was important for peer volunteers' acceptability (Atif *et al*., 2016). The peer volunteers felt that engagement with the training and supervision had improved their own psychosocial wellbeing, social status and job opportunities. Many of the peer volunteers went on to find work with other local maternal and child health agencies and two peer volunteers were elected as lady-counsellors (a political appointment) for their areas. Studies in other LMICs, where laywomen were deployed to deliver community maternal health care, have reported similar findings. They had improved empowerment and knowledge (Alcock *et al*., 2009), enhanced social status and better mobility within their communities (Nankunda *et al*., 2007; Alcock *et al*., 2009). In addition to the training and supervision, another contributing factor to the peers' competency was the simplicity of the intervention (Atif *et al*., 2016). The intervention was adapted to make it deliverable by local laywoman with at least 10 years of schooling and no work experience (Atif *et al*., 2017). The key messages were reinforced during supervisions and emphasis was laid on empathetic listening.

Some of the barriers to the training included peer volunteers' lack of prior training experience. This was addressed through using narratives they could relate to, using culturally appropriate activities and drawing on their personal experiences. Other barriers included training impinging on peer volunteers' household commitments and need for more refresher trainings. The refresher trainings were incorporated into on-going supervisions through revising the intervention contents and practising delivery of the sessions through role-plays. The timings for the training and supervision sessions were planned to avoid clash with their routine household commitments. For the local trainers, a challenge to conduct monthly supervisions was lack of facilities at the BHUs. BHUs located in the rural areas are underfunded and lack in basic amenities (Nishtar, 2006), making it difficult for trainers to conduct supervisions in an ideal setting. In one study area, the majority of the supervisions were conducted outside the BHU due to room unavailability. However, the relationship between the trainers and peer volunteers, built over time, helped them to conduct effective supervisions despite all its challenges.

The master trainer, located outside Pakistan, conducted the supervisions via Skype. The technology-assisted supervision, in the context of this study, was the most feasible alternative to face-to-face supervision. In order to ensure effective supervision using technology, the roles of the supervisor and supervisees were clarified at the outset, plans were put in place to manage technical issues, additional time was set aside in case needed for trouble shooting issues with technology and emphasis was laid on supervisory relationship. These are consistent with practical tips suggested by Martin *et al*. (2017) based on their review of the literature on use of technology for clinical supervision. Focus on the supervisory relationship is highlighted as one of the critical factor for effective and high quality supervision. In this study, supervisor–supervisee relationship was built upon the mutual trust and respect, the foundation of which was laid during face-to-face training. Overall, distant-based supervision proved effective and majority of the session went undisrupted. However, a few sessions were postponed or cancelled due to internet issues.

In rural Pakistan, the work opportunities for women outside the domestic arena are almost non-existent. The enthusiasm of peer volunteers is indicated by the retention of the majority, and supportive supervision played a part in maintaining it. The programme managed to retain most of their peer volunteers, working voluntarily, over the period of 3 years. This is consistent with the evidence from literature suggesting both pre-service and in-service training a key factor in reducing turnover (Kohrt *et al*., 2015). However, volunteer run programmes have high turnover and therefore need to have systems in place to rapidly recruit and train new volunteers. The current programme encouraged peer volunteers intending to leave, to identify other peer volunteers with similar characteristics to replace them. This worked successfully, as the new peer volunteers quickly achieved the required competencies and took over the job of those who left.

In this study, the quality of the intervention and the peer volunteers' competency were maintained through direct observation of the session and using the Therapy and Competency Checklist. According to Fairburn & Cooper (2011) therapy quality refers to the extent to which a therapy was delivered well enough for it to achieve its expected effects and therapist competency refers to the therapist knowledge and skills required to deliver it. Both aspects, in this study, were assured through direct observation and evaluation of the treatment sessions and evaluating the role-plays during trainings and supervisions. Evidence from the literature indicates quality control methods utilising active training strategies and review of observational treatment session data are more effective than the methods that rely on passive learning (Fairburn & Cooper, 2011; Garland & Schoenwald, 2013; Southam-Gerow & McLeod, 2013). The direct observations allowed evaluation of the peer volunteers' adherence to the intervention and their knowledge and ability to implement it. The evaluations were scored on the Quality and Competency Checklist specifically developed for this purpose. The checklist was informed from an established instrument that has been used in similar studies in the region (Kohrt *et al*., 2015). However, the validation or reliability of the tool has not been evaluated, and therefore the assessed scores might not be indicative of actual skills. One indirect indicator of the tool's validity was that peer competencies evaluated improved over time and with supervision. This was expected, and captured by the tool, indicating it was sensitive to such changes. Another limitation is that the trainers rated their peer volunteers' competencies and conducted the focus groups (albeit a different set of peers from the ones trained and supervised by them) and this may have introduced an element of bias in the study. The study was conducted in one rural Pakistani setting, where a high degree of collaboration between the study team, primary health system, peer volunteers and the community was established. The findings should therefore be generalised with caution.

Overall, our findings indicate that this model can be effectively deployed to train and supervise local peer volunteers who achieve satisfactory competency in the skills required to deliver the programme. The model has the potential to address the paucity of mental health specialists, by utilising the specialists' time and skills most effectively.

In conclusion, the study demonstrates that lay-workers such as peer volunteers can be trained and supervised to deliver a psychological intervention using a cascaded model, thus addressing the barrier of scarcity of specialist trainers and supervisors.
